# S100B and LDH as early prognostic markers for response and overall survival in melanoma patients treated with anti-PD-1 or combined anti-PD-1 plus anti-CTLA-4 antibodies

**DOI:** 10.1038/s41416-018-0167-x

**Published:** 2018-06-28

**Authors:** Nikolaus B Wagner, Andrea Forschner, Ulrike Leiter, Claus Garbe, Thomas K Eigentler

**Affiliations:** Department of Dermatology, University Hospital Tuebingen, Liebermeisterstrasse 25, 72076 Tuebingen, Germany

**Keywords:** Melanoma, Prognostic markers, Tumour biomarkers

## Abstract

**Background:**

Immunotherapy with PD-1 antibodies has greatly increased prognosis of patients with advanced melanoma. Identifying biomarkers that predict overall survival (OS) and response to immunotherapy is important.

**Methods:**

OS and best overall response according to RECIST version 1.1 were analysed, and S100B and lactate dehydrogenase (LDH) serum levels were assessed retrospectively in 152 patients treated with anti-PD-1, and in 86 patients treated with anti-PD-1 plus anti-CTLA-4 antibodies at University Hospital Tuebingen, Germany.

**Results:**

In the pembrolizumab group, patients with elevated baseline S100B or LDH exhibited significantly impaired OS compared with patients with normal S100B (1-year OS: 51.1% vs 83.1%, log-rank *P* < .0001) and normal LDH (1-year OS: 44.4% vs 80.8%, *P* = .00022), respectively. LDH increases of >25% and S100B increases of >145% compared to baseline were significantly associated with impaired OS (both *P* < .0001). In patients treated with ipilimumab and nivolumab, baseline S100B and increasing S100B levels of >145% as well as baseline LDH were associated with impaired OS (*P* < .0001, *P* = .00060, and *P* = .0050, respectively), whereas increasing LDH of >25% was not (*P* = .64).

**Conclusions:**

S100B could serve as a strong baseline marker for OS in melanoma patients receiving anti-PD-1 therapy. Rising S100B levels during the first weeks of therapy could help guide treatment decisions.

## Introduction

Anti-programmed death receptor-1 (anti-PD-1) antibodies alone or in combination with cytotoxic T-lymphocyte antigen-4 (CTLA-4) antibodies have improved the prognosis of metastatic melanoma substantially and have been shown to induce long-lasting partial or complete responses in melanoma patients.^1–3^ However, primary or acquired resistance against immune checkpoint inhibitors occurs in 50–60% of the patients.^4^ Thus, identifying predictive markers for response to immunotherapy with PD-1 or CTLA-4 antibodies can be beneficial in guiding treatment decisions.

Although a plethora of putative biomarkers and clinical markers for response and survival have been proposed in the recent past, most of these markers are not suitable for monitoring the clinical course of melanoma patients during immune checkpoint blockade with PD-1 and/or CTLA-4 antibodies due to costs or methodological complexity.

Serum lactate dehydrogenase (LDH) is a well-known biomarker for metastatic melanoma patients and since 2009 a part of the revised melanoma staging guidelines from the American joint committee on cancer (AJCC).^5–8^ In recent trials with immune checkpoint inhibitors elevated baseline LDH had consistently been shown to correlate with poor survival and poor response rates.^3,9^ Moreover, a retrospective study on 66 patients receiving the PD-1 antibodies nivolumab or pembrolizumab reported that increases in LDH levels between start of treatment and the first staging were associated with poor response and diminished overall survival (OS).^10^

In comparison to the abundantly distributed and unspecific cytosolic enzyme LDH, the calcium-binding, acidic cytoplasmic protein S100B (also known as S-100B or S-100β) is considered a specific and reliable immunohistochemical marker in malignant melanoma.^11–14^ Moreover, elevated serum S100B had been shown to be associated with poor survival in metastatic melanoma patients treated in the chemotherapy era.^6,15–20^ The determination of S100B is recommended in the current German guideline as part of the regular tumour surveillance from stage IB as biomarker for progression.^21^ In patients receiving ipilimumab, elevated baseline S100B as well as at week 3 and week 6 during treatment was associated with impaired OS and S100B levels increased 12 weeks after starting ipilimumab in patients who deceased after treatment.^22,23^ Although S100B levels correlated significantly with progression-free survival (PFS) and melanoma-specific survival, they were not found to correlate with overall response.^24^ To the best of our knowledge, for patients receiving PD-1 antibodies or the combined immunotherapy with PD-1 and CTLA-4 antibodies data on serum S100B are still unknown.

In this study, we asked whether (i) baseline levels of LDH and S100B are prognostic biomarkers in patients treated with pembrolizumab or nivolumab plus ipilimumab, and (ii) whether relative changes in these markers within the first weeks of treatment indicate response and correlate with OS.

## Materials and methods

This single-centre retrospective study included patients with unresectable stage III or stage IV melanoma treated with the PD-1 antibody pembrolizumab (cohort 1) or with combined immunotherapy with the CTLA-4 antibody ipilimumab and the PD-1 antibody nivolumab (cohort 2) at the University Hospital Tuebingen, Germany between June 2014 and July 2017. Patients were included if they had known baseline LDH and S100B, and if they had received at least one infusion with the immune checkpoint inhibitors. Patient data, clinical variables, and laboratory values were obtained from electronic patient records and were pseudonymised. The study was approved by the local Research Ethics Board (reference number 436/2017BO2). All patients had given their written informed consent for data collection within the Central Malignant Melanoma Registry.

### Treatment and response assessment

Patients received either pembrolizumab (2 mg kg^−1^ every 3 weeks) or ipilimumab (3 mg kg^−1^) plus nivolumab (1 mg kg^−1^) every 3 weeks for four cycles followed by nivolumab (3 mg kg^−1^ every 2 weeks). Response was evaluated by computed tomography (CT), magnetic resonance imaging (MRI), or positron emission tomography (PET-CT). Clinical response was assessed according to Response Evaluation Criteria in Solid Tumours (RECIST) version 1.1.^25^ Baseline serum LDH and serum S100B were measured not more than 7 days before administration of anti-PD-1 and/or CTLA-4 antibodies.

### Statistical analysis

Response according to RECIST criteria 1.1 and OS defined as the time from starting anti-PD-1 or anti-CTLA-4 + anti-PD-1 therapy until death due to any cause or end of follow-up were explored in all patients. Patients were stratified based on baseline LDH values (below or equal to 1.5 × the upper limit of normal (ULN) compared above 1.5 × ULN) and baseline S100B values (below or equal to 0.3 µg/l compared above 0.3 µg/l). The cut-off point for S100B was chosen according to previous reports.^15^ For LDH, the cut-off point was chosen arbitrarily, to the end that the number of patients with elevated LDH being similar to the number of patients with elevated S100B and with the aim of achieving significant differences in both cohorts. The cut-off was neither set to 1 × ULN nor to 2.5 × ULN due to non-significant results in one of the two cohorts each. Moreover, the 2.5 × ULN cut-off identified very low numbers of patients under risk (10 patients in cohort 1, and 9 patients in cohort 2).

In addition to the baseline biomarker analyses, we assessed early changes in LDH and S100B levels with the aim of investigating whether changes in serum LDH and serum S100B could predict response and OS before the first radiological assessment. For this purpose, only patients with at least one early post-baseline LDH and S100B value were considered. Biomarker measurements were considered early post-baseline if done within the first 6 weeks after the first cycle. Relative increase or decrease of successive LDH or S100B values from baseline were calculated. For Kaplan–Meier survival analyses, patients were categorised based on cut-off points for the relative changes in LDH or S100B levels which were calculated based on cohort 1 data by means of a *P*-value minimising approach.^26^ The cut-off points for relative changes of LDH and S100B were +25% and +145%, respectively. For the analyses of cohort 2, the same cut-off points were used.

Univariate analyses of OS were carried out using Kaplan–Meier estimator. *P*-values were calculated using two-sided log-rank test. Hazard ratios were calculated using Cox regression analysis. Multivariate survival analyses were assessed utilising multivariate Cox regression. Two-sided Mann–Whitney U-test was used to compare means of continuous variables among groups. Categorical variables were compared using two-sided Chi-squared test. Throughout all analyses, *P*-values < 0.05 were considered statistically significant. All analyses were performed using R version 3.4.0 and the ‘survival’ package (R Core Team, 2017).

## Results

### Patient characteristics

238 patients with advanced melanoma were included in our study (152 patients in cohort 1, and 86 patients in cohort 2). Detailed clinical characteristics are summarised in Table 1. Most patients had stage M1c disease (76% in cohort 1, 86% in cohort 2) and had visceral metastases (65% in cohort 1, 84% in cohort 2). Around 30% of the patients in both cohorts had central nervous system (CNS) metastasis. Liver metastasis was present in 30% of the patients in cohort 1 and in 49% in cohort 2. 39% and 43% of the patients were treatment-naïve, respectively. Median follow-up from start of immunotherapy was 9.9 months (interquartile range [IQR] 4.8–15.7 months) in cohort 1, and 6.4 months (IQR 3.2–10.6 months) in cohort 2.

**Table 1:** Descriptive statistics

	Cohort 1 (*N*=152)	Cohort 2 (*N*=86)
*Treatment regimen*		
anti-PD-1	152 (100.0%)	0 (0%)
anti-PD-1 + anti-CTLA-4	0 (0%)	86 (100.0%)
*Number of cycles administered*		
One	12 (7.9%)	9 (10.5%)
Two	12 (7.9%)	14 (16.3%)
Three	12 (7.9%)	20 (23.3%)
Four (or more in cohort 2)	4 (2.6%)	43 (50.0%)
Five (or more in cohort 1)	112 (73.7%)	
*Sex*		
Female	64 (42.1%)	36 (41.9%)
Male	88 (57.9%)	50 (58.1%)
*Age*		
≤ 60 years	50 (32.9%)	34 (39.5%)
> 60 years	102 (67.1%)	52 (60.5%)
BRAF mutation		
No	99 (65.1%)	60 (69.8%)
Yes	50 (32.9%)	22 (25.6%)
Unknown	3 (2.0%)	4 (4.7%)
*Number of organs involved before treatment*		
One	20 (13.2%)	12 (14.0%)
Two	51 (33.6%)	10 (11.6%)
Three	32 (21.1%)	28 (32.6%)
Four	20 (13.2%)	10 (11.6%)
Five	14 (9.2%)	11 (12.8%)
Six	8 (5.3%)	5 (5.8%)
Seven or more	6 (3.9%)	9 (10.5%)
Unknown	1 (0.7%)	1 (1.2%)
*AJCC M stage (AJCC classification 2009)*		
M0	10 (6.6%)	4 (4.7%)
M1a	6 (3.9%)	1 (1.2%)
M1b	21 (13.8%)	7 (8.1%)
M1c	115 (75.7%)	74 (86.0%)
*Visceral metastasis*		
No	54 (35.5%)	14 (16.3%)
Yes	98 (64.5%)	72 (83.7%)
*CNS metastasis*		
No	106 (69.7%)	61 (70.9%)
Yes	46 (30.3%)	25 (29.1%)
*Liver metastasis*		
No	107 (70.4%)	44 (51.2%)
Yes	45 (29.6%)	42 (48.8%)
*Number of prior treatment regimens*		
Zero	59 (38.8%)	37 (43.0%)
One	41 (27.0%)	23 (26.7%)
Two	27 (17.8%)	10 (11.6%)
Three	22 (14.5%)	11 (12.8%)
Four	3 (2.0%)	5 (5.8%)
Unknown	0 (0%)	0 (0%)

*AJCC* American Joint Committee on Cancer, *CNS* central nervous system, CTLA-4 = cytotoxic T-lymphocyte-associated protein 4, *N* number of patients, *PD-1* programmed cell death protein 1

### Association of baseline LDH and S100B with survival

**Fig. 1:**
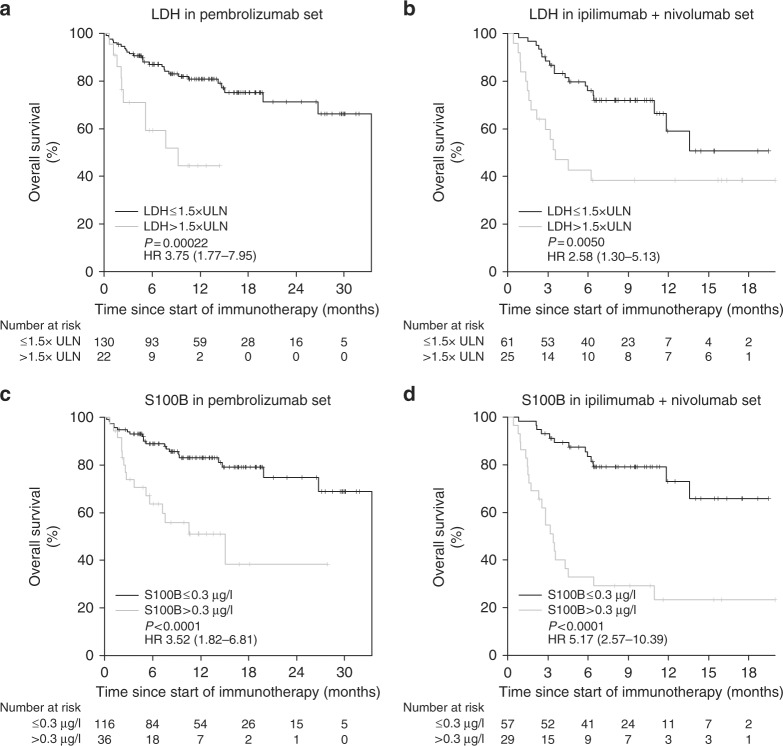
Overall survival in (**a**) group 1 and (**b**) group 2 according to baseline LDH, and in (**c**) group 1 and (**d**) group 2 according to baseline S100B. HR hazard ratio, LDH lactate dehydrogenase, *P* *P*-value, ULN upper limit of normal

Survival analysis at 1 year after initiation of immunotherapy showed a clear correlation of death with high biomarker levels (Supplementary Figure S1). Univariate analysis of OS revealed significantly shortened OS in patients with elevated lactate dehydrogenase LDH > 1.5 × upper limit of normal (ULN) compared with patients with LDH ≤ 1.5 × ULN in cohort 1 (hazard ratio (HR) 3.75, 95% confidence interval (CI) 1.77–7.95, *P* = .00022) and in cohort 2 (HR 2.58, 95% CI 1.30–5.13, *P* = .0050) (Fig. 1a, b). Patients with S100B levels > 0.3 µg/l also presented with significantly shortened OS in cohort 1 (HR 3.52, 95% CI 1.82–6.81, *P* < .0001) as well as in cohort 2 (HR 5.17, 95% CI 2.57–10.39, *P* < .0001) (Fig. 1c, d).

Multivariate Cox regression analysis of OS in the pembrolizumab treated patients of cohort 1 including S100B, LDH, and the well-known prognostic factor visceral metastasis (model 1) revealed S100B (HR 2.54, 95% CI 1.20–5.37, *P* = .014), LDH (HR 2.39, 95% CI 1.02–5.59, *P* = .045) and visceral metastasis (HR 4.04, 95% CI 1.56–10.45, *P* = .0039) as independent prognostic factors (Table 2). Multivariate model 2 included S100B, LDH, visceral metastasis as well as brain metastasis, another well-known prognostic factor associated with survival. In model 2, S100B (HR 2.93, 95% CI 1.40–6.17, *P* = .0045) was independently associated with OS, whilst LDH (HR 2.06, 95% CI 0.89–4.78, *P* = .09), CNS metastasis (HR 2.15, 95% CI 0.98–4.70, *P* = .06) and visceral metastasis (HR 2.60, 95% CI 0.88–7.65, *P* = .08) were not (Table 2). In multivariate analysis of OS in the ipilimumab + nivolumab treated patients of cohort 2 S100B was independently associated with OS in both models (model 1: HR 6.97, 95% CI 2.87–16.89, *P* < .0001; model 2: HR 7.29, 95% CI 2.97–17.89, *P* < .0001) (Table 3).

**Table 2:** Multivariate analysis of overall survival in cohort 1 (pembrolizumab)

				Model 1			Model 2		
Factor	*N*	%	% Dead	HR	95% CI	*P*	HR	95% CI	*P*
*S100B*									
≤ 0.3 µg/l	116	76.3	19.0	1			1		
> 0.3 µg/l	36	23.7	44.4	2.54	1.20–5.37	0.014	2.93	1.40–6.17	0.005
*LDH*									
≤1.5xULN	130	85.5	21.5	1			1		
>1.5xULN	22	14.5	45.5	2.39	1.02–5.59	0.045	2.06	0.89–4.78	0.09
*Visceral metastasis*									
No	54	35.5	9.3	1			1		
Yes	98	64.5	33.7	4.04	1.56–10.45	0.004	2.60	0.88–7.65	0.08
*CNS metastasis*									
No	106	69.7	16.0				1		
Yes	46	30.3	45.7				2.15	0.98–4.70	0.06
*Origin*									
Cutaneous	119	78.3	26.1	1			1		
Occult	17	11.2	23.5	1.01	0.34–3.00	0.98	1.15	0.39–3.41	0.79
Mucosal	8	5.3	25.0	0.52	0.12–2.23	0.38	0.72	0.16–3.20	0.67
Uveal	8	5.3	12.5	0.51	0.07–3.87	0.51	0.86	0.10–7.14	0.89

*CI* confidence interval, *CNS* central nervous system, *HR* hazard ratio, *LDH* lactate dehydrogenase, *N* number of patients, *P* *P*-value

**Table 3:** Multivariate analysis of overall survival in cohort 2 (ipilimumab + nivolumab)

				Model 1			Model 2		
Factor	*N*	%	% Dead	HR	95% CI	*P*	HR	95% CI	*P*
*S100B*									
≤0.3 µg/l	57	66.3	22.8	1			1		
>0.3 µg/l	29	33.7	72.4	6.97	2.87–16.89	<0.0001	7.29	2.97–17.89	<0.0001
*LDH*									
≤1.5xULN	61	70.9	31.1	1			1		
>1.5xULN	25	29.1	60.0	1.29	0.58–2.86	0.53	1.22	0.55–2.73	0.63
*Visceral metastasis*									
No	14	16.3	28.6	1			1		
Yes	72	83.7	41.7	0.98	0.33–2.95	0.97	0.76	0.24–2.42	0.64
*CNS metastasis*									
No	61	70.9	36.1				1		
Yes	25	29.1	48.0				1.82	0.81–4.09	0.15
*Origin*									
Cutaneous	59	68.6	35.6	1			1		
Occult	16	18.6	50.0	2.74	1.00–7.53	0.051	3.02	1.10–8.26	0.032
Mucosal	4	4.7	75.0	1.08	0.30–3.83	0.91	0.82	0.21–3.10	0.77
Uveal	7	8.1	28.6	2.07	0.41–10.56	0.38	2.65	0.50–14.15	0.25

*CI* confidence interval, *CNS* central nervous system, *HR* hazard ratio, *LDH* lactate dehydrogenase, *N* number of patients, *P* *P*-value

### Changes in LDH and S100B levels between start of treatment and first staging

**Fig. 2:**
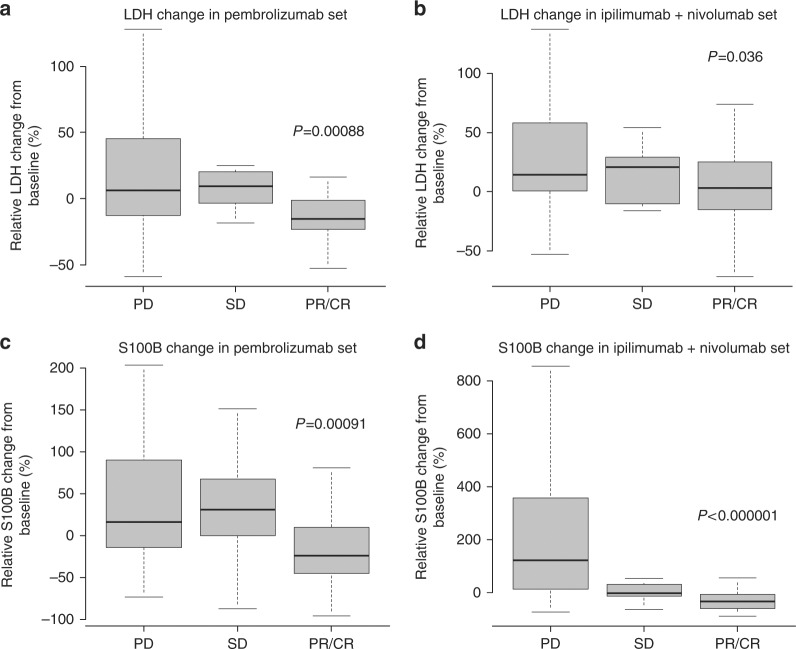
Association between changes in LDH or S100B after the first two cycles of immunotherapy and tumour response in the (a and c) pembrolizumab cohort, and in the (b and d) ipilimumab + nivolumab cohort. CR complete response, LDH lactate dehydrogenase, *P* *P*-value, PD progressive disease, PR partial response, SD stable disease

For this analysis, only patients with LDH and S100B values within the first 6 weeks after the first cycle of immunotherapy were included. Figure 2 shows the association between the changes in LDH or S100B and first objective response determined by using RECIST version 1.1. Patients treated with pembrolizumab who achieved a partial or complete response (PR/CR) had a marked reduction of LDH compared with their baseline value (median: −15.6%; ICR: −23.1% to −1.3%) as well as of S100B (median: −24.1%, ICR: −43.5% to 7.7%) (Fig. 2a, c). In contrast, patients showing progressive disease (PD) at the first staging more frequently had an increase in their LDH values (median: 6.2%, ICR: −12.8% to 44.5%) and S100B values (median: 16.3%, ICR: −14.1% to 89.2%). These changes in LDH and S100B levels during the first 6 weeks of treatment were significantly different between responders and patients with PD (LDH: *P* = .00088, S100B: *P* = .00091). Between cycle 3 and cycle 5, S100B levels continued to rise in patients with PD (mean delta between S100B at cycle 3 and at cycle 5: 1.202 ± 0.733 [mean ± standard error of the mean] µg/l vs. -0.041 ± 0.025 µg/l, *P* = .0014) (Supplementary Figure S2). In patients treated with the combined immune checkpoint inhibition, changes in LDH significantly differed between responders (PR/CR; median: 3.2%, IQR: −15.3% to 25.4%) and non-responders (PD; median: 14.2%, IQR: 0.4–58.3%) (*P* = .036, Fig. 2b). However, strongly decreasing S100B levels were observed in responders to ipilimumab plus nivolumab (PR/CR; median: -32.0%, IQR: −60.2% to −6.7%), and median S100B levels significantly increased in patients with PD (median: 121.7%, IQR: 12.8% to 357.3%) (*P* < .000001, Fig. 2d). In patients previously treated with anti-PD-1 monotherapy, courses of S100B levels differed prominently between responders and non-responders (Supplementary Figure S3). Responders exhibited decreasing S100B levels after switching from anti-PD-1 monotherapy to combined immune checkpoint inhibition whereas S100B levels continued to rise in non-responders.

**Fig. 3:**
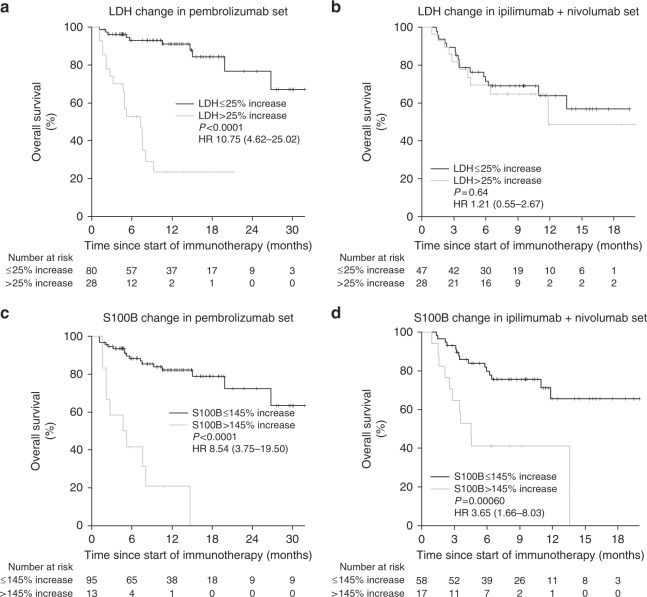
Overall survival in (**a**) group 1 and (**b**) group 2 according to early changes in LDH, and in (**c**) group 1 and (**d**) group 2 according to change in S100B. HR hazard ratio, LDH lactate dehydrogenase, *P* *P*-value

Using a threshold of 25% increase and 145% increase for LDH and S100B, respectively, increases in LDH (HR 10.75, 95% CI 4.62–25.02, *P* < .0001) and S100B (HR 8.54, 95% CI 3.75–19.50, *P* < .0001) both were strongly associated with lower OS in patient treated with pembrolizumab (Fig. 3a, c). These findings were consistently found independent of their baseline biomarker level (Supplementary Figures S4 and S5). In patients treated with the combined immunotherapy with ipilimumab and nivolumab, LDH increases were not associated with impaired OS (HR 1.21, 95% CI 0.55–2.67, *P* = .64), whereas S100B increases were again significantly associated with impaired OS (HR 3.65, 95% CI 1.66–8.03, *P* = .00060) (Fig. 3b, d, and Supplementary Figures S4 and S5).

## Discussion

Our study shows that patients with elevated levels of LDH > 1.5 × ULN and S100B > 0.3 µg/l exhibit significantly diminished OS compared with those patients with lower levels of LDH and S100B when treated with the PD-1 antibody pembrolizumab. Moreover, we show that an increase in LDH and S100B during the first weeks of treatment can predict disease progression several weeks prior to the first CT/MRI-based staging and that such increasing biomarker levels are also associated with impaired OS. In patients treated with the combined immunotherapy (ipilimumab plus nivolumab), our data provide evidence that measurement of S100B is useful for predicting OS, whereas LDH seems to be less predictive in these patients.

Elevated serum LDH and S100B are well-known markers for poor outcome of metastatic melanoma patients in the pre-immune checkpoint inhibitor era.^5,6,17–20,27–29^ However, only LDH is part of the AJCC melanoma staging guideline for metastatic melanoma patients.^7^ The era of immune checkpoint inhibitors began with the approval of the CTLA-4 antibody ipilimumab which turned out to be superior to chemotherapeutic agents.^30,31^ The PD-1 antibodies nivolumab and pembrolizumab achieve even better response rates and long-term OS rates with fewer side effects as ipilimumab.^1,3^ Nivolumab combined with ipilimumab resulted in even longer PFS and OS than nivolumab alone, but toxicity is significantly increased with 55–59% grade 3 or 4 treatment-related adverse events compared to 16–21% in the nivolumab treatment arm.^3,32^ Prognostic markers predicting response and OS to immune checkpoint inhibitors are desirable to select patients who might benefit from combined immunotherapy or even MAPK-targeted therapy with BRAF and MEK inhibitors.

Many studies have already shown that in patients treated with ipilimumab, an increased baseline LDH is independently associated with poor survival.^33–35^ Increased baseline LDH was also reported to be associated with diminished OS and lower objective response rates in patients receiving nivolumab or pembrolizumab.^9,10,36^ In those patients with elevated baseline LDH, further increasing LDH levels before the first staging have also been shown to be predictive for shortened OS.^10^ However, Diem et al. included only 29 patients with elevated baseline LDH for the analysis of increasing LDH levels. Our data support these findings with elevated baseline LDH > 1.5 × ULN and further increasing LDH levels being significantly associated with poor OS in a cohort of 152 patients treated with pembrolizumab. In addition, we show that increasing LDH levels are not only a useful predictor of OS in patients with elevated baseline LDH, but also in patients with normal baseline LDH.

Concerning S100B, only three reports investigated the possible impact of elevated serum S100B on poor outcome in patients treated with ipilimumab.^23,24,37^ To the best of our knowledge, there are no reports on S100B in patients treated with PD-1 antibodies so far. Our data are the first to demonstrate the prognostic potential of S100B in these patients. Moreover, we show in this large cohort of 152 patients that S100B can be a powerful and independent marker for OS, superior to LDH and the other well-known factors visceral and CNS metastasis. The results for increasing serum S100B levels during the first weeks of PD-1 blockade were comparable to the results for increasing LDH concerning the association with objective response and OS.

The results for the cohort of 86 patients receiving ipilimumab in combination with nivolumab were unexpected. Elevated baseline LDH was associated with OS, but early changes in LDH levels during the first weeks of treatment were not. Even more clearly, patients showing partial (PR) or complete response (CR) did not have significantly decreasing LDH levels during the first weeks of treatment. Interestingly, Postow et al. reported similar response rates in patients with LDH below or above ULN (63.2% vs. 53.3%).^38^ A large phase I dose-escalation study also stated a promising 3-year OS rate of 61% in patients with elevated LDH > ULN and ≤ 2 × ULN compared to 70% in patients with normal baseline LDH.^39^ However, the so far largest phase 3 trial comparing nivolumab plus ipilimumab vs. nivolumab vs. ipilimumab with over 300 patients in each treatment arm (CheckMate 067) reported a 66% 3-year OS rate in patients with LDH ≤ ULN and only 44% and 31% 3-year OS rates in patients with LDH > ULN and LDH > 2 × ULN, respectively.^3^ Of note, the study population in this clinical trial considerably differs from the ‘real world’ setting in the present study. The percentage of patients with elevated baseline LDH was 36% compared to 49% in our study. Other significant differences between the CheckMate 067 trial and our study included 41% vs. 49% patients ≥ 65 years, 59% vs. 87% stage M1c, 28% vs. 13% patients with only one lesion site, 4% vs. 29% brain metastasis. The circumstance of stable or even increasing LDH levels in responders to combined immunotherapy raises the question whether the high frequency of immune-related adverse events could be responsible for this issue. Rising levels of LDH can be found for instance in patients developing autoimmune hepatitis or colitis. Afzal et al. reported on a case of a patient with uveal melanoma developing autoimmune hepatitis with concomitant continuously rising LDH while responding on ipilimumab plus nivolumab.^40^ Therefore, for patients receiving combined checkpoint inhibition with ipilimumab and nivolumab, LDH cannot be certainly considered as the main prognostic marker, at least in the setting of early biomarker changes during therapy. Additional markers for response and survival are required. Compared with the modest results for LDH in the present study, both baseline S100B > 0.3 µg/l as well as increases in S100B levels of more than 145% were significantly associated with poor OS in our data. Furthermore, the results were highly significant for S100B increases in patients with PD and decreases in patients with PR or CR, respectively.

We are aware of limitations of our study. The retrospective design makes it susceptible for a patient selection bias. However, we included all consecutive patients at our centre receiving pembrolizumab or combined nivolumab plus ipilimumab. Thereby, there was probably even less selection than in most clinical trials where patients with ECOG status > 1 or brain metastasis are either excluded or underrepresented. Another limitation of our study is the lack of information about the PD-L1 status in the tumour tissue which may be a prognostic and predictive marker for OS and response, especially in patients receiving the combination of nivolumab plus ipilimumab.^2,3,32^

In conclusion, we postulate LDH and S100B to be useful and easily measurable markers at baseline and during treatment with pembrolizumab. They could be utilised to identify patients who might benefit from an earlier staging evaluation after 1.5–2 months compared to the usual 3 months. Those patients with increasing biomarker levels during the first weeks of immune checkpoint inhibition could therefore be forwarded earlier to a rescue therapy regimen. Although our data suggest a slight superiority of S100B compared to LDH, the definite assessment and comparison of the two biomarkers should be subject to larger prospective trials. Further prospective trials are required to confirm our results and to deepen the understanding of these biomarkers in guiding treatment decisions before and during the course of treatment.

## Electronic supplementary material

Supplemental Figure S1

Supplemental Figure S2

Supplemental Figure S3

Supplemental Figure S4

Supplemental Figure S5

## Availability of data and material

The data that support the findings of this study are available from the corresponding author upon reasonable request.
