# Supplementary material for: S100B and LDH as early prognostic markers for response and overall survival in melanoma patients treated with anti-PD-1 or combined anti-PD-1 plus anti-CTLA-4 antibodies
Source: Br J Cancer. 2018 Jun 28;119(3):339–46. doi: 10.1038/s41416-018-0167-x (PMC6070917; doi:10.1038/s41416-018-0167-x)
Supplement: Supplementary file 1 — Supplemental Figure S1 [file 41416_2018_167_MOESM1_ESM.pptx]

## Slide 1
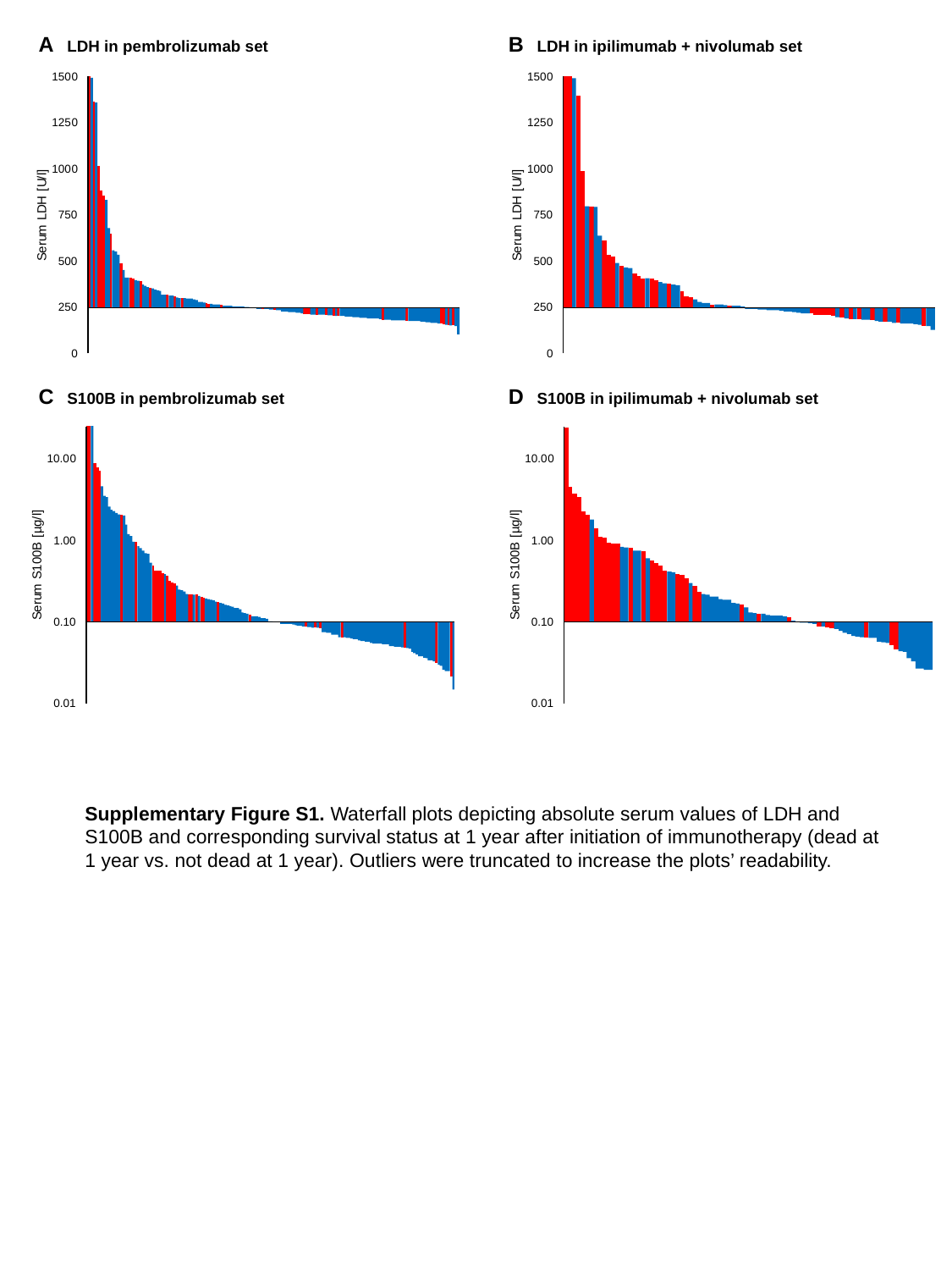

A LDH in pembrolizumab set
B LDH in ipilimumab + nivolumab set
C S100B in pembrolizumab set
D S100B in ipilimumab + nivolumab set
Supplementary Figure S1. Waterfall plots depicting absolute serum values of LDH and S100B and corresponding survival status at 1 year after initiation of immunotherapy (dead at 1 year vs. not dead at 1 year). Outliers were truncated to increase the plots’ readability.
