# Supplementary material for: S100B and LDH as early prognostic markers for response and overall survival in melanoma patients treated with anti-PD-1 or combined anti-PD-1 plus anti-CTLA-4 antibodies
Source: Br J Cancer. 2018 Jun 28;119(3):339–46. doi: 10.1038/s41416-018-0167-x (PMC6070917; doi:10.1038/s41416-018-0167-x)
Supplement: Supplementary file 2 — Supplemental Figure S2 [file 41416_2018_167_MOESM2_ESM.pptx]

## Slide 1
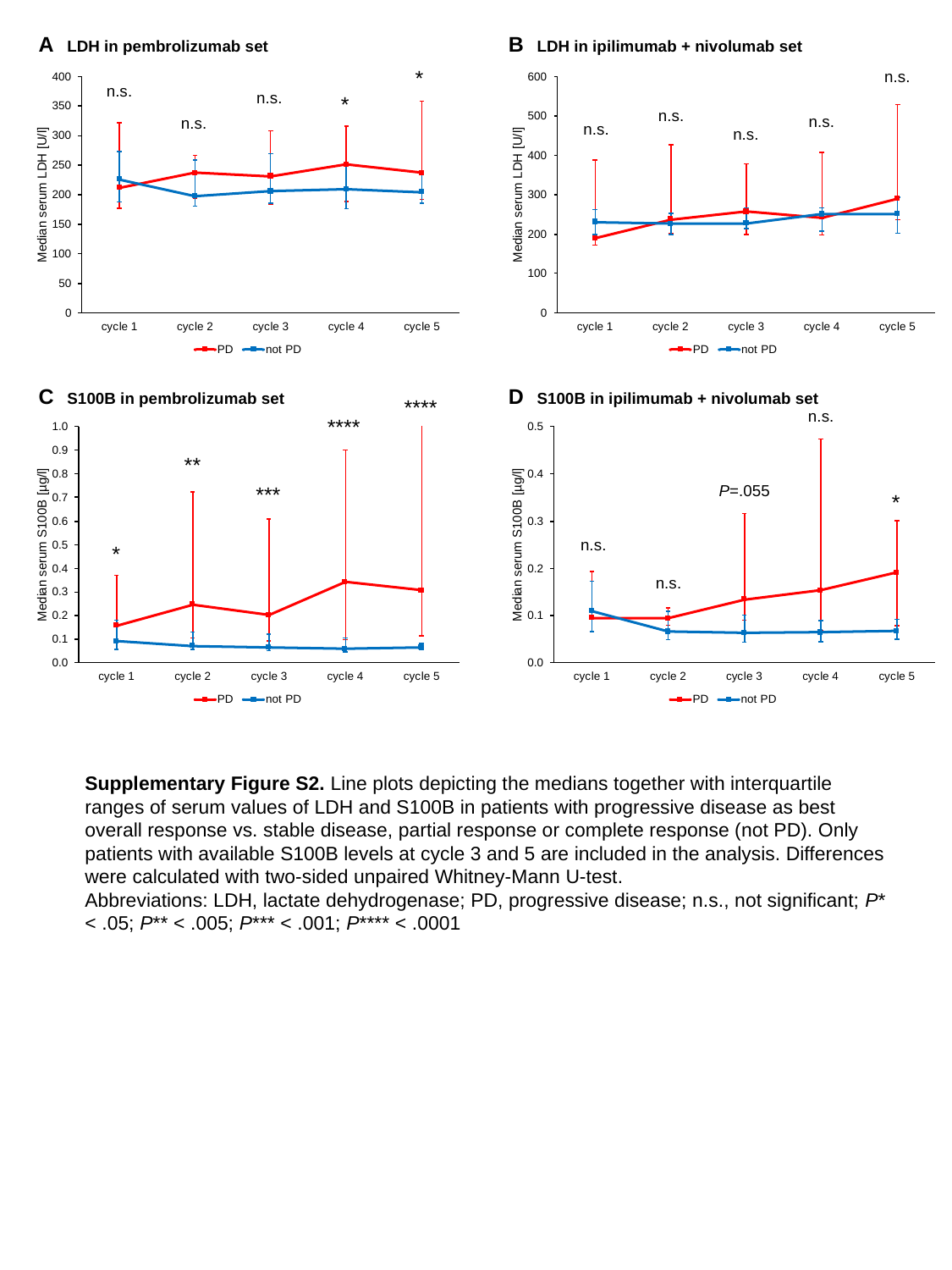

A LDH in pembrolizumab set
B LDH in ipilimumab + nivolumab set
*
n.s.
n.s.
n.s.
*
n.s.
n.s.
n.s.
n.s.
n.s.
C S100B in pembrolizumab set
D S100B in ipilimumab + nivolumab set
****
n.s.
****
**
P=.055
***
*
n.s.
*
n.s.
Supplementary Figure S2. Line plots depicting the medians together with interquartile ranges of serum values of LDH and S100B in patients with progressive disease as best overall response vs. stable disease, partial response or complete response (not PD). Only patients with available S100B levels at cycle 3 and 5 are included in the analysis. Differences were calculated with two-sided unpaired Whitney-Mann U-test.
Abbreviations: LDH, lactate dehydrogenase; PD, progressive disease; n.s., not significant; P* < .05; P** < .005; P*** < .001; P**** < .0001
