# Supplementary material for: S100B and LDH as early prognostic markers for response and overall survival in melanoma patients treated with anti-PD-1 or combined anti-PD-1 plus anti-CTLA-4 antibodies
Source: Br J Cancer. 2018 Jun 28;119(3):339–46. doi: 10.1038/s41416-018-0167-x (PMC6070917; doi:10.1038/s41416-018-0167-x)
Supplement: Supplementary file 3 — Supplemental Figure S3 [file 41416_2018_167_MOESM3_ESM.pptx]

## Slide 1
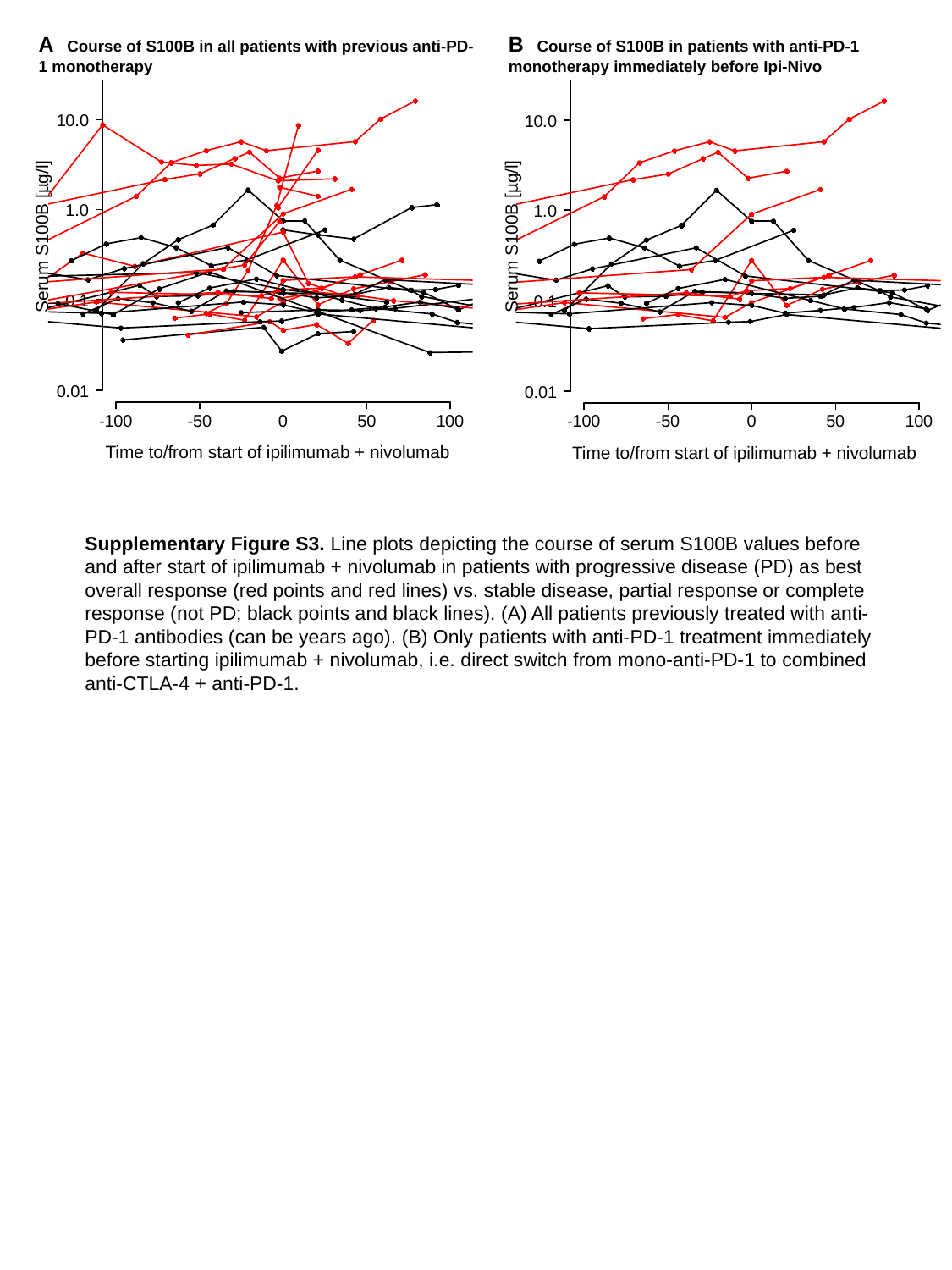

A Course of S100B in all patients with previous anti-PD-1 monotherapy
B Course of S100B in patients with anti-PD-1 monotherapy immediately before Ipi-Nivo
Serum S100B [µg/l]
Serum S100B [µg/l]
Time to/from start of ipilimumab + nivolumab
Time to/from start of ipilimumab + nivolumab
Supplementary Figure S3. Line plots depicting the course of serum S100B values before and after start of ipilimumab + nivolumab in patients with progressive disease (PD) as best overall response (red points and red lines) vs. stable disease, partial response or complete response (not PD; black points and black lines). (A) All patients previously treated with anti-PD-1 antibodies (can be years ago). (B) Only patients with anti-PD-1 treatment immediately before starting ipilimumab + nivolumab, i.e. direct switch from mono-anti-PD-1 to combined anti-CTLA-4 + anti-PD-1.
