# Supplementary material for: S100B and LDH as early prognostic markers for response and overall survival in melanoma patients treated with anti-PD-1 or combined anti-PD-1 plus anti-CTLA-4 antibodies
Source: Br J Cancer. 2018 Jun 28;119(3):339–46. doi: 10.1038/s41416-018-0167-x (PMC6070917; doi:10.1038/s41416-018-0167-x)
Supplement: Supplementary file 4 — Supplemental Figure S4 [file 41416_2018_167_MOESM4_ESM.pptx]

## Slide 1
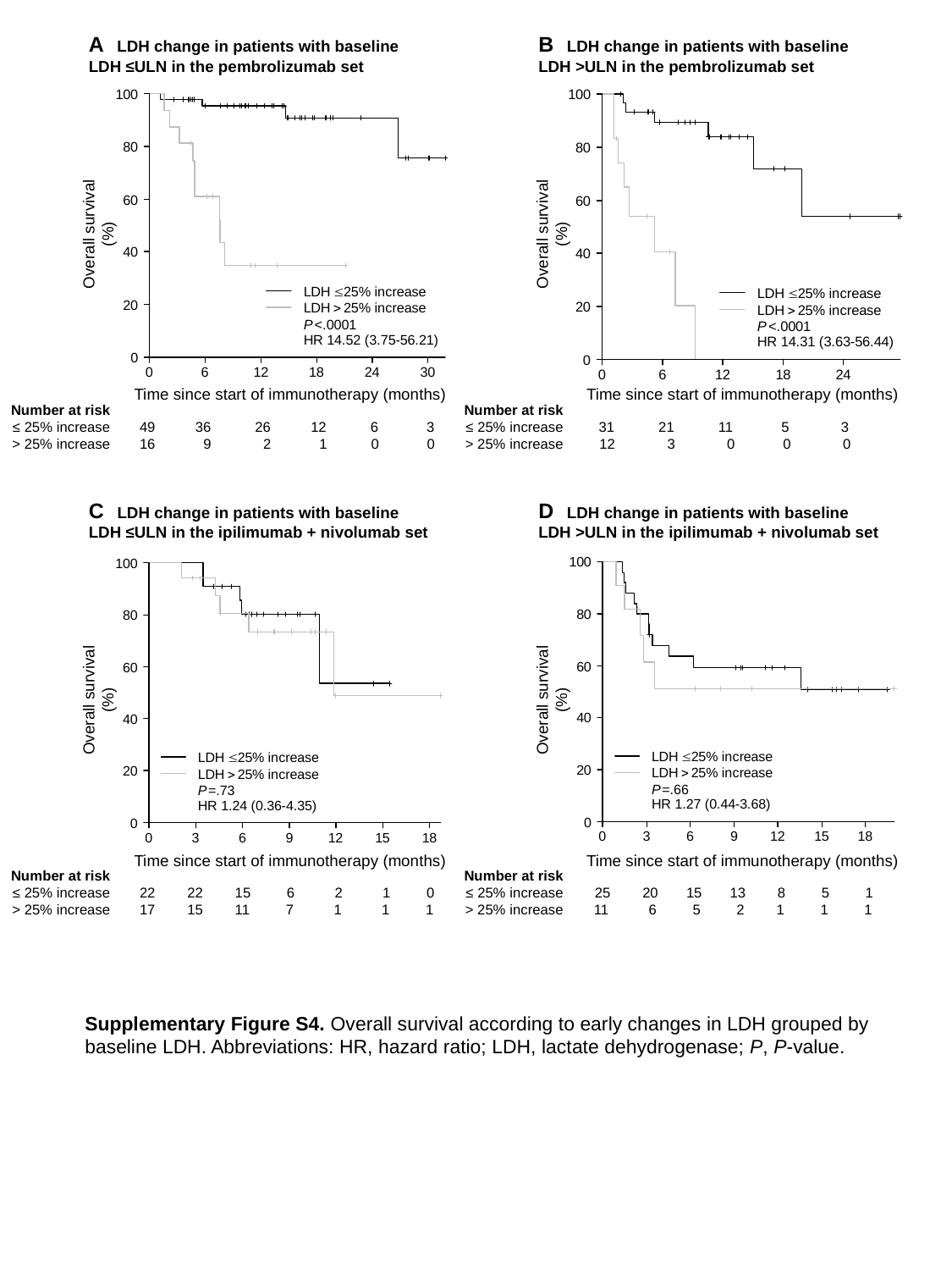

A LDH change in patients with baseline LDH ≤ULN in the pembrolizumab set
B LDH change in patients with baseline
LDH >ULN in the pembrolizumab set
Overall survival (%)
Overall survival (%)
Time since start of immunotherapy (months)
Time since start of immunotherapy (months)
Number at risk
≤ 25% increase
> 25% increase
Number at risk
≤ 25% increase
> 25% increase
 49 36 26 12 6 3 31 21 11 5 3
 16 9 2 1 0 0 12 3 0 0 0
C LDH change in patients with baseline
LDH ≤ULN in the ipilimumab + nivolumab set
D LDH change in patients with baseline
LDH >ULN in the ipilimumab + nivolumab set
Overall survival (%)
Overall survival (%)
Time since start of immunotherapy (months)
Time since start of immunotherapy (months)
Number at risk
≤ 25% increase
> 25% increase
Number at risk
≤ 25% increase
> 25% increase
 22 22 15 6 2 1 0 25 20 15 13 8 5 1
 17 15 11 7 1 1 1 11 6 5 2 1 1 1
Supplementary Figure S4. Overall survival according to early changes in LDH grouped by baseline LDH. Abbreviations: HR, hazard ratio; LDH, lactate dehydrogenase; P, P-value.
