# Supplementary material for: S100B and LDH as early prognostic markers for response and overall survival in melanoma patients treated with anti-PD-1 or combined anti-PD-1 plus anti-CTLA-4 antibodies
Source: Br J Cancer. 2018 Jun 28;119(3):339–46. doi: 10.1038/s41416-018-0167-x (PMC6070917; doi:10.1038/s41416-018-0167-x)
Supplement: Supplementary file 5 — Supplemental Figure S5 [file 41416_2018_167_MOESM5_ESM.pptx]

## Slide 1
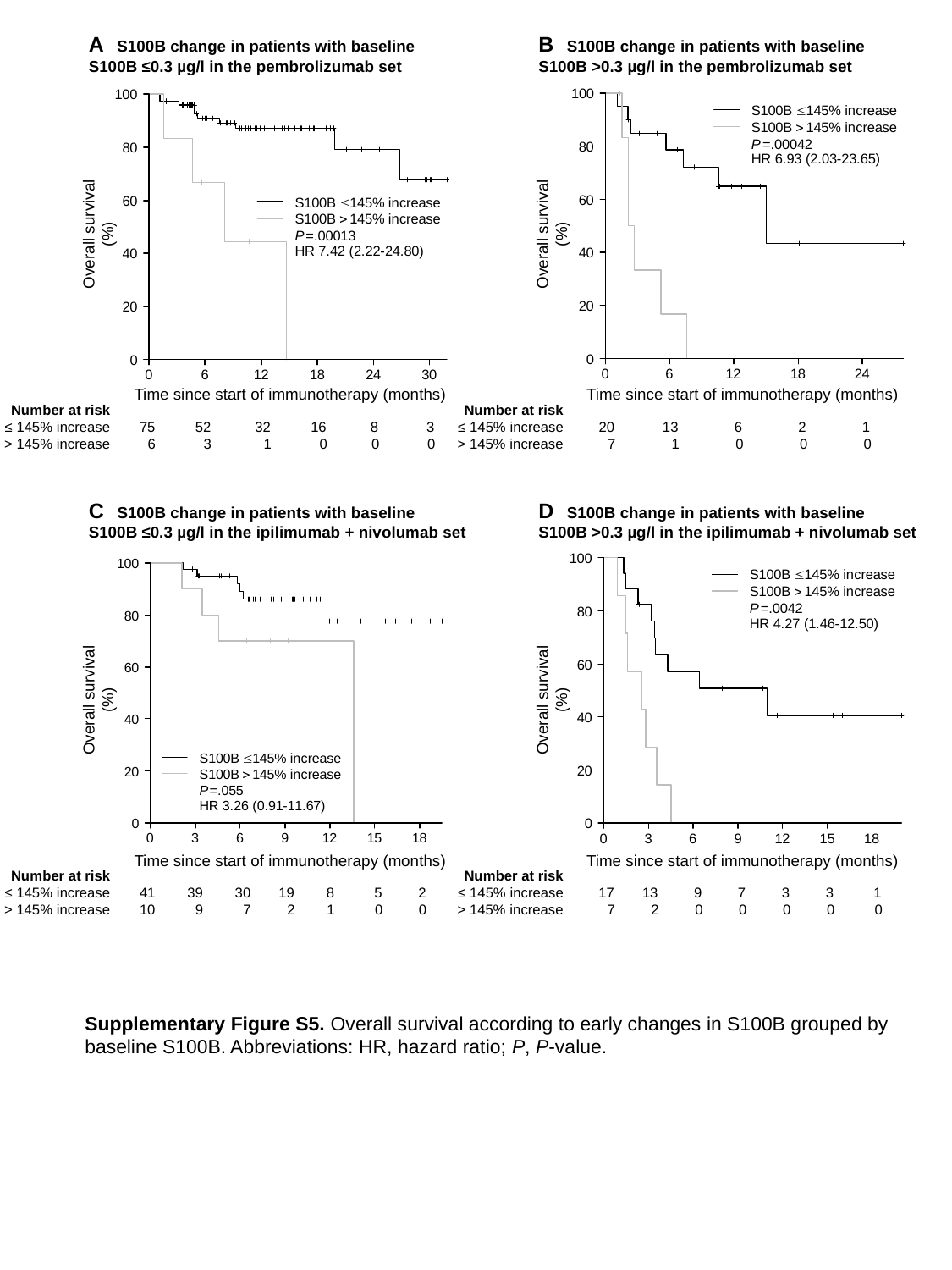

A S100B change in patients with baseline S100B ≤0.3 µg/l in the pembrolizumab set
B S100B change in patients with baseline
S100B >0.3 µg/l in the pembrolizumab set
Overall survival (%)
Overall survival (%)
Time since start of immunotherapy (months)
Time since start of immunotherapy (months)
Number at risk
≤ 145% increase
> 145% increase
Number at risk
≤ 145% increase
> 145% increase
 75 52 32 16 8 3 20 13 6 2 1
 6 3 1 0 0 0 7 1 0 0 0
C S100B change in patients with baseline
S100B ≤0.3 µg/l in the ipilimumab + nivolumab set
D S100B change in patients with baseline
S100B >0.3 µg/l in the ipilimumab + nivolumab set
Overall survival (%)
Overall survival (%)
Time since start of immunotherapy (months)
Time since start of immunotherapy (months)
Number at risk
≤ 145% increase
> 145% increase
Number at risk
≤ 145% increase
> 145% increase
 41 39 30 19 8 5 2 17 13 9 7 3 3 1
 10 9 7 2 1 0 0 7 2 0 0 0 0 0
Supplementary Figure S5. Overall survival according to early changes in S100B grouped by baseline S100B. Abbreviations: HR, hazard ratio; P, P-value.
